# Enhancing surfactin production by using systematic CRISPRi repression to screen amino acid biosynthesis genes in *Bacillus subtilis*

**DOI:** 10.1186/s12934-019-1139-4

**Published:** 2019-05-23

**Authors:** Congya Wang, Yingxiu Cao, Yongping Wang, Liming Sun, Hao Song

**Affiliations:** 1 Frontier Science Center for Synthetic Biology and Key Laboratory of Systems Bioengineering (MOE), School of Chemical Engineering and Technology, Tianjin University, Tianjin, 300350 People’s Republic of China; 2 Petrochemical Research Institute, PetroChina Company Limited, Beijing, 102206 China

**Keywords:** Surfactin, CRISPR interference, Amino acids, *Bacillus subtilis*

## Abstract

**Background:**

Surfactin is a cyclic lipopeptide that is of great industrial use owing to its extraordinary surfactant power and antimicrobial, antiviral, and antitumor activities. Surfactin is synthesized by a condensation reaction in microbes, which uses fatty acids and four kinds of amino acids (l-glutamate, l-aspartate, l-leucine and l-valine) as precursors. Surfactin biosynthesis could be improved by increasing the supply of fatty acids; however, the effect of the regulation of amino acid metabolism on surfactin production was not yet clear.

**Results:**

In this study, we aimed to improve surfactin production in *B. subtilis* by repressing the genes on the branch metabolic pathways of amino acid biosynthesis using CRISPRi technology. First, 20 genes were inhibited individually, resulting in 2.5- to 627-fold decreases in transcriptional level as determined by RT-qPCR. Among the 20 recombinant strains, 16 strains obtained higher surfactin titres than that produced by the parent BS168NU-Sd strain (the surfactin production of BS168NU-Sd with only dCas9 but no sgRNA expression was 0.17 g/L). In particular, the strains in which the *yrpC*, *racE* or *murC* genes were inhibited individually produced 0.54, 0.41, or 0.42 g/L surfactin, respectively. All three genes are related to the metabolism of l-glutamate, whose acylation is the first step in the surfactin condensation reaction. Furthermore, these three genes were repressed in combination, and the strain with co-inhibition of *yrpC* and *racE* produced 0.75 g/L surfactin, which was 4.69-fold higher than that of the parent strain. In addition, the inhibition of *bkdAA* and *bkdAB,* which are related to the metabolism of l-leucine and l-valine, not only improved surfactin production but also increased the proportion of the C_14_ isoform.

**Conclusions:**

This study, to the best of our knowledge for the first time, systematically probed the regulatory effect of increasing the supply of amino acids on surfactin production. It provided an effective strategy and a new perspective for systematic studies on surfactin and other amino acid-derived chemicals.

**Electronic supplementary material:**

The online version of this article (10.1186/s12934-019-1139-4) contains supplementary material, which is available to authorized users.

## Background

**Fig. 1:**
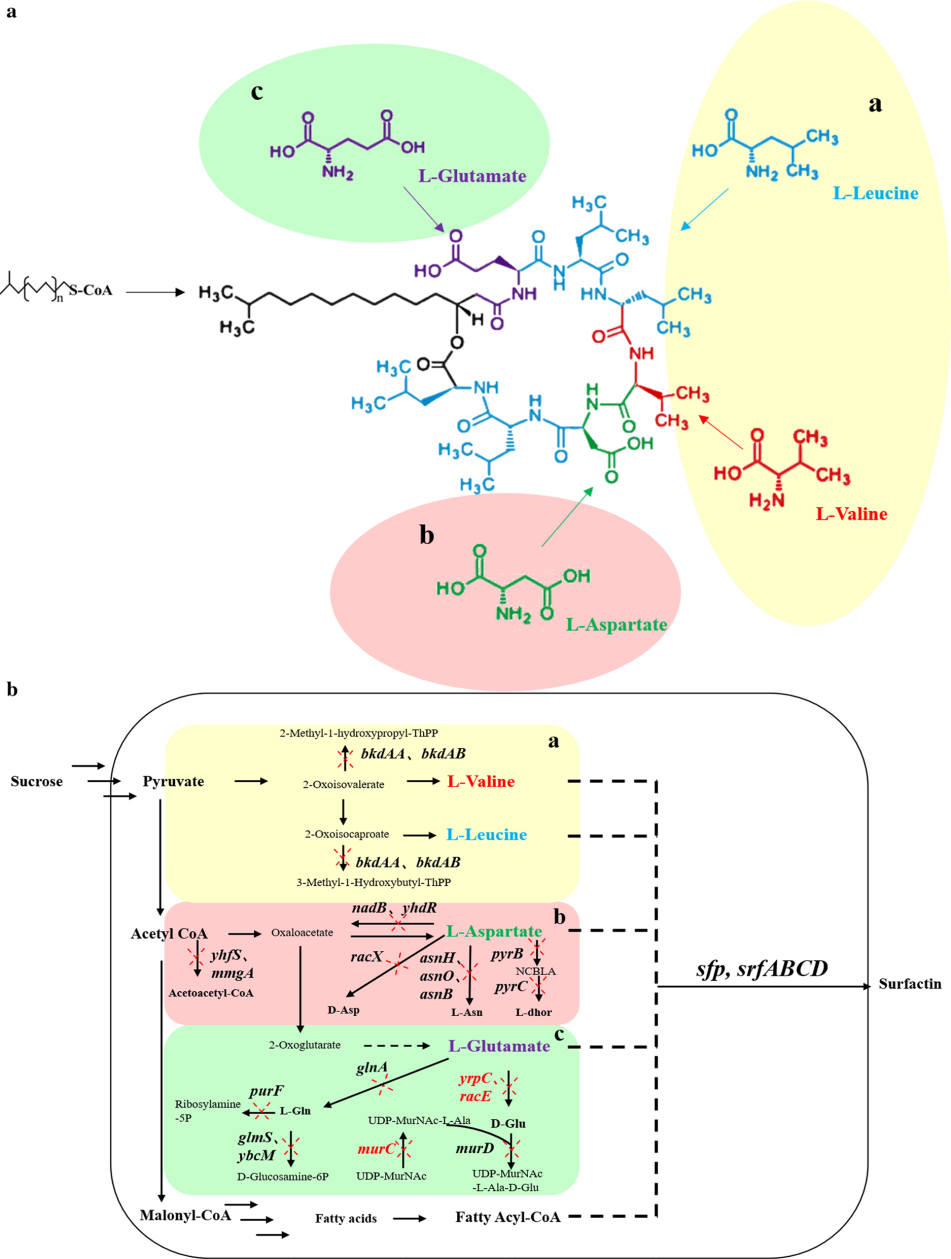
Schematic of surfactin biosynthesis in *B. subtilis*. **a** The chemical structure of surfactin. Surfactin is a cyclic lipopeptide consisting of a C_12_–C_19_ β-hydroxy fatty acid chain and a cyclic heptapeptide consisting of four kinds of amino acids. Fatty acid chain: black; l-glutamate: purple; l-aspartate: green; l-leucine: blue; and l-valine: red. **b** The de novo biosynthesis of surfactin in *B. subtilis*. The synthesis of surfactin can be divided into three parts: the biosynthesis of fatty acids and the activation of fatty acid chains to form fatty acyl-CoA; the biosynthesis of the four kinds of amino acids; and the assembly of surfactin. The amino acids are assembled onto the fatty acyl-CoA successively via surfactin synthase. Different coloured shadings indicate different modules, including the l-valine and l-leucine metabolic modules (a. light yellow), the l-aspartate metabolic module (b. light cameo brown), and the l-glutamate metabolic module (c. light green). d-Glu: d-glutamate; d-Asp: d-aspartate; l-Asn: l-asparagine; l-dhor: l-dihydroorotate; NCBLA: *N*-carbamoyl-l-aspartate; l-Gln: l-glutamine; UDP-MurNAc-l-Ala: *N*-acetylmuramoyl-l-alanine; and UDP-MurNAc-l-Ala-d-Glu: *N*-acetylmuramoyl-l-alanine-d-glutamate

Surfactin is a lipopeptide that contains two acidic amino acid residues (glutamate and aspartate), five nonpolar amino acid residues (leucine and valine) and a C_12_–C_19_ β-hydroxy fatty acid chain [1, 2], as shown in Fig. 1a. Surfactin is an efficient biosurfactant and has potent antimicrobial, antiviral, and antitumor activities, which are widely used in oil recovery, biopesticides, food processing, cosmetics, and pharmaceuticals [3, 4]. Surfactin can be biosynthesized by many natural *Bacillus subtilis* strains. Despite many efforts to enhance surfactin production [5–7], however, the large-scale production and industrial application of surfactin remained restricted by its low production [8–10].

The synthesis of surfactin can be mainly divided into three parts (Fig. 1b): the biosynthesis of fatty acids that are activated to form fatty acyl-CoA by fatty acyl-CoA ligase [11]; the biosynthesis of four kinds of amino acids; and the assembly of surfactin, in which seven amino acids are assembled successively onto fatty acyl-CoA via surfactin synthase, which is encoded by the *srfA* operon and regulated by cell density signal and phosphopantetheinyl transferase (PPTase) Sfp. Previous studies mainly focused on the third part, and surfactin biosynthesis was enhanced by increasing the expression level of *srfA*. For example, Jiao et al. [8] and Sun et al. [12] enhanced surfactin production by replacing the original constitutive promoter of *srfA* (P*srfA*) with stronger inducible Pg3 or Pspac promoters. Others achieved *srfA* overexpression by upregulating the quorum sensing system *ComQXPA* [11, 13–15] or downregulating the negative factors [13, 14, 16–19]. Dhali et al. [20]. enhanced the production to 1556 ± 123 mg of surfactin per g dry weight of cell biomass by knocking out the global regulation factor *codY*, which had negative effects on *srfA* expression. Regarding the precursor supply, recent research suggested that increasing the supply of fatty acid precursors could greatly enhance the surfactin titre to ~ 4.9 g/L in a flask using modified synthetic medium by overexpressing the genes involved in the fatty acid biosynthesis pathway [17]. On the other hand, amino acids are also essential precursors for surfactin biosynthesis. Thus, increasing the amino acid supply might also have positive effects on surfactin production and could be achieved by decreasing the metabolism flux in the branch pathways for amino acid biosynthesis [21–23]. However, no such study had previously been performed.

Recent advances in the field of synthetic biology are expediting our ability to regulate metabolic pathways and enhance the synthesis of target chemicals. In particular, the advent of clustered regularly interspersed short palindromic repeat interference (CRISPRi), which requires only the dCas9 enzyme, which is defunct in endo-nucleolytic activity because of point mutations in the RuvC and HNH domains, and a custom single guide RNA (sgRNA) that conveys the DNA binding specificity to dCas9 [24], enabling the rapid repression of gene transcription. The CRISPRi system has been successfully used to improve the biosynthesis of target chemicals by decreasing the metabolic flux of branch pathways. Lv et al. [25] downregulated the expression of *sad*, *sucC*, *sucD*, *sdhA*, and *sdhB* simultaneously via CRISPRi to regulate the carbon flux from succinate synthesis pathways to 4-hydroxybutyrate (4HB) biosynthesis and increased the 4HB content in the poly((3HB-co-4HB)) to 18.4 mol%. Wu et al. [23] silenced the expression of *ppsA*, *eno*, *adhE*, *mdh*, *fumC*, *sdhA*, *sucC* and *citE* simultaneously by CRISPRi and enhanced the malonyl-CoA concentration 2.3-fold. CRISPRi was also used to improve the production of polyhydroxyalkanoates [26], fatty alcohols [27], shikimic acid [28], *O*-methylated anthocyanin [29], and poly-β-hydroxybutyrate [30].

In this study, we tried to enhance surfactin production by increasing the supply of amino acids. First, we incorporated the surfactin biosynthesis pathway into BS168NU by expressing the exogenous *sfp* gene (BS168NU-S). Second, we constructed an effective CRISPRi system, as demonstrated by transcriptional repression measured by RT-qPCR. Third, the expression of 20 genes, which were selected from the branch metabolic pathways of amino acids, was perturbed individually by CRISPRi. Among the 20 recombinant strains, 16 recombinant strains gained increased surfactin production. In particular, the strains in which *yrpC, racE* or *murC* was inhibited enhanced surfactin production to 0.54, 0.41 or 0.42 g/L, respectively. Then, the three genes were further repressed in combination. The results indicated that the strains with co-inhibition of *yrpC* and *racE* obtained the highest production, which was directly related to l-glutamate metabolism, whose acylation was the first step of surfactin assembly. This work revealed that engineering the amino acid metabolism is an efficient strategy to enhance the production of surfactin.

## Materials and methods

### Genes, strains and plasmids

The *sfp* gene derived from *Bacillus amyloliquefaciens* DSM7 was synthesized by Genewiz (Suzhou, China), and the sequence is shown in Additional file 1: Table S1. All the wild-type and recombinant bacterial strains are listed in Table 1, and all the plasmids used in this work are listed in Table 2. *E. coli* Trans T1 was purchased from TransGen Biotech (Beijing, China) and used for gene cloning and plasmid construction. BS168 NU, constructed by our laboratory, was the starting strain for the construction of the strains for surfactin production. The pJMP1 and pJMP2 plasmids were purchased from Beijing Zhongyuan Heju Economic and Trade Co., Ltd.

**Table 1:** Strains used in this study

Name	Genotype	Source/references
*E. coli* Trans T1	F-φ80(*lac*Z)ΔM15Δ*lac*X74*hsd*R(r_k_^−^, m_k_^+^)Δ*rec*A1398*end*A1*ton*A	TransGen Biotech
*B. subtilis* 168	trpC2	Laboratory stock
BS168NU	trpC2, Δ*araR*::P*ara*-*neo*, Δ*upp*	Laboratory stock
MK3-MEP2-m	BS168NU, Δ*yxlA*::PlapS-*menA*, Δ*ydeO*::P43-dxr-DN*-cat-*araR*	Laboratory stock
BS168NU-S	BS168NU, Δ*ydeO*::P43-sfp	This work
BS168NU-Sd	BS168NU-S derivate, Δ*lacA*::Pxyl-dCaS9	This work
mmgA	BS168NU-Sd derivate, Δ*amyE*::P*veg*-*sgRNA*^*mmgA*^	This work
yhfS	BS168NU-Sd derivate, Δ*amyE*::P*veg*-*sgRNA*^*yhfS*^	This work
asnB	BS168NU-Sd derivate, Δ*amyE*::P*veg*-*sgRNA*^*asnB*^	This work
asnH	BS168NU-Sd derivate, Δ*amyE*::P*veg*-*sgRNA*^*asnH*^	This work
asnO	BS168NU-Sd derivate, Δ*amyE*::P*veg*-*sgRNA*^*asnO*^	This work
nadB	BS168NU-Sd derivate, Δ*amyE*::P*veg*-*sgRNA*^*nadB*^	This work
yhdR	BS168NU-Sd derivate, Δ*amyE*::P*veg*-*sgRNA*^*yhdR*^	This work
racX	BS168NU-Sd derivate, Δ*amyE*::P*veg*-*sgRNA*^*racX*^	This work
pyrB	BS168NU-Sd derivate, Δ*amyE*::P*veg*-*sgRNA*^*pyrB*^	This work
pyrC	BS168NU-Sd derivate, Δ*amyE*::P*veg*-*sgRNA*^*pyrC*^	This work
purF	BS168NU-Sd derivate, Δ*amyE*::P*veg*-*sgRNA*^*purF*^	This work
glnA	BS168NU-Sd derivate, Δ*amyE*::P*veg*-*sgRNA*^*GlnA*^	This work
yrpC	BS168NU-Sd derivate, Δ*amyE*::P*veg*-*sgRNA*^*yrpC*^	This work
racE	BS168NU-Sd derivate, Δ*amyE*::P*veg*-*sgRNA*^*racE*^	This work
glmS	BS168NU-Sd derivate, Δ*amyE*::P*veg*-*sgRNA*^*glmS*^	This work
ybcM	BS168NU-Sd derivate, Δ*amyE*::P*veg*-*sgRNA*^*ybcM*^	This work
murD	BS168NU-Sd derivate, Δ*amyE*::P*veg*-*sgRNA*^*murD*^	This work
murC	BS168NU-Sd derivate, Δ*amyE*::P*veg*-*sgRNA*^*murC*^	This work
bkdAA	BS168NU-Sd derivate, Δ*amyE*::P*veg*-*sgRNA*^*bkdAA*^	This work
bkdAB	BS168NU-Sd derivate, Δ*amyE*::P*veg*-*sgRNA*^*bkdAB*^	This work
H1	BS168NU-Sd derivate, Δ*amyE*::P*veg*-*sgRNA*^*yrpC*^, P*veg*-*sgRNA*^*racE*^	This work
H2	BS168NU-Sd derivate, Δ*amyE*::P*veg*-*sgRNA*^*yrpC*^, P*veg*-*sgRNA*^*murC*^	This work
H3	BS168NU-Sd derivate, Δ*amyE*::P*veg*-*sgRNA*^*racE*^, P*veg*-*sgRNA*^*murC*^	This work
H4	BS168NU-Sd derivate, Δ*amyE*::P*veg*-*sgRNA*^*yrpC*^, P*veg*-*sgRNA*^*racE*^, P*veg*-*sgRNA*^*murC*^	This work

**Table 2:** Plasmids used in this study

Plasmids	Characteristics	Source/references
pJMP1	P*xyl*-*dCaS9*, ErmR	[37]
pJMP2	P*veg*-*sgRNA*^*RR1*^, CmR	[37]
pmmgA	pJMP2 derivate, P*veg*-*sgRNA*^*mmgA*^	This work
pyhfS	pJMP2 derivate, P*veg*-*sgRNA*^*yhfS*^	This work
pasnB	pJMP2 derivate, P*veg*-*sgRNA*^*asnB*^	This work
pasnH	pJMP2 derivate, P*veg*-*sgRNA*^*asnH*^	This work
pasnO	pJMP2 derivate, P*veg*-*sgRNA*^*asnO*^	This work
pnadB	pJMP2 derivate, P*veg*-*sgRNA*^*nadB*^	This work
pyhdR	pJMP2 derivate, P*veg*-*sgRNA*^*yhdR*^	This work
pracX	pJMP2 derivate, P*veg*-*sgRNA*^*racX*^	This work
ppyrB	pJMP2 derivate, P*veg*-*sgRNA*^*pyrB*^	This work
ppyrC	pJMP2 derivate, P*veg*-*sgRNA*^*pyrC*^	This work
ppurF	pJMP2 derivate, P*veg*-*sgRNA*^*purF*^	This work
pglnA	pJMP2 derivate, P*veg*-*sgRNA*^*glnA*^	This work
pyrpC	pJMP2 derivate, P*veg*-*sgRNA*^*yrpC*^	This work
pracE	pJMP2 derivate, P*veg*-*sgRNA*^*racE*^	This work
pglmS	pJMP2 derivate, P*veg*-*sgRNA*^*glmS*^	This work
pybcM	pJMP2 derivate, P*veg*-*sgRNA*^*ybcM*^	This work
pmurD	pJMP2 derivate, P*veg*-*sgRNA*^*murD*^	This work
pmurC	pJMP2 derivate, P*veg*-*sgRNA*^*murC*^	This work
pbkdAA	pJMP2 derivate, P*veg*-*sgRNA*^*bkdAA*^	This work
pbkdAB	pJMP2 derivate, P*veg*-*sgRNA*^*bkdAB*^	This work
pH1	pJMP2 derivate, P*veg*-*sgRNA*^*yrpC*^, P*veg*-*sgRNA*^*racE*^	This work
pH2	pJMP2 derivate, P*veg*-*sgRNA*^*yrpC*^, P*veg*-*sgRNA*^*murC*^	This work
pH3	pJMP2 derivate, P*veg*-*sgRNA*^*racE*^, P*veg*-*sgRNA*^*murC*^	This work
pH4	pJMP2 derivate, P*veg*-*sgRNA*^*yrpC*^, P*veg*-*sgRNA*^*racE*^, P*veg*-*sgRNA*^*murC*^	This work

### Media and bacterial growth

Luria–Bertani medium (LB, 10 g/L tryptone, 5 g/L yeast extract, and 10 g/L NaCl) was used for *E. coli* and *B. subtilis* growth, and solid agar was used for the growth of *E. coli* and *B. subtilis* colonies.

The following materials were used to obtain competent cells: 10× Spizizen (10× Spizizen: 20 g/L (NH4)_2_SO_4_, 183 g/L K_2_HPO_4_, 60 g/L KH_2_PO_4_, 12 g/L sodium citrate), GMI medium (5 mL of GMI: 500 μL of 10× Spizizen, 100 μL of 2% casein acids hydrolysate, 100 μL of 5% yeast, 100 μL of 40% glucose, 5 μL of 20% MgSO_4_∙H_2_O, 50 μL of 0.5% l-tryptophan and 4.14 mL of H_2_O) and GMII medium (5 mL of GMII: 500 μL of 10× Spizizen, 50 μL of 2% casein acids hydrolysate, 100 μL of 40% glucose, 40 μL of 20% MgSO_4_∙H_2_O, and 4.31 mL of H_2_O).

The surfactin production fermentation medium of the *B. subtilis* strains comprised 70 g/L sucrose, 1 g/L yeast extract, 25 g/L NaNO_3_, 0.333 g/L KH_2_PO_4_, 1 g/L Na_2_HPO_4_∙12H_2_O, 0.15 g/L MgSO_4_∙7H_2_O, 7.5 mg/L CaCl_2_, 6 mg/L MnSO_4_∙H_2_O, and 6 mg/L FeSO_4_∙7H_2_O (pH 7.0). The seed culture solution of *B. subtilis* strains was grown for 12 h in LB medium. On the next day, 500 μL of seed culture solution was inoculated into a test tube containing 5 mL of fermentation medium with an appropriate concentration of xylose inducer. The cell densities and surfactin concentrations were measured simultaneously at the required time points. All strains were cultured at 37 °C with shaking at 220 rpm. Whenever required, antibiotics were added to the medium at the following concentrations: 100 μg/mL ampicillin for *E. coli*; 6 μg/mL chloramphenicol, 10 μg/mL erythromycin and/or 15 μg/mL neomycin for *B. subtilis*.

### Competent cells and translation

Competent cells were obtained as described by Anagnostopoulos and Spizizen [31]. A single colony of the receptor strain was picked up in a test tube with 5 mL of GMI medium and cultured for 14–16 h at 37 °C with shaking at 200 rpm. Then, 500 μL of broth was transferred into another test tube containing 4.5 mL of GMI medium and cultured for 4.5 h at 37 °C with shaking at 200 rpm to achieve mid-late logarithmic growth of the bacteria. Then, 750 μL of culture was transferred into another test tube containing 4.25 mL of GMII medium and cultured for 1.5 h at 37 °C with shaking at 240 rpm to obtain competent cells.

Then, 0.5 to 2 μg of DNA fragment was added to 1 mL of competent cells and mixed well and the cells were then cultured for 1.5 h at 37 °C with shaking at 200 rpm. If plasmids were added, they were mixed and placed 1 h at 37 °C statically. Then, the cells were cultured for 1–1.5 h at 37 °C with shaking 240 rpm. Then, the EP tube was centrifuged for 2 min at 13,000 rpm. After removing 900 μL of the supernatant, the cells were suspended in the remaining 100 μL of supernatant, and the transformants were coated on a LB solid plate for screening. After 12–18 h of incubation at 37 °C, single colonies were obtained for further screening of transformants.

### Marker-free gene modification

The method of marker-free gene modification used here for *sfp* gene expression was derived from Liu et al. [32]. In the chromosome BS168NU, the *araR* locus was replaced by the counter-selective marker cassette (P*ara*-*neo*) through double crossover homologous recombination, enabling the colonies of BS168NU to grow on an Nm-resistance plate. Then, the selective marker cassette (CR), which was constituted by the Cm-resistance gene (*cat*) and the *araR* gene, and the recombination fragment (upstream fragment U, downstream fragment G and homologous recombination fragment D) were integrated into the target locus in the order UDCRG. The expression of the Nm-resistance gene (*neo*) was repressed by the transcriptional repressor AraR, so the colonies were selected on a Cm-resistant plate. Finally, the selective marker cassette was evicted by single-crossover with homologous recombination fragment D, and the colonies were selected on an Nm-resistance plate.

### sgRNA design [33]

The first CCN was identified as the protospacer adjacent motif (PAM) sequence in the non-template strand, and the following 12 bp were regarded as the seed region. To avoid off-target effects, we searched the genome of *B. subtilis* 168 for the 15 bp specificity region consisting of the 12 bp ‘seed’ region of the sgRNA and 3 bp (CCN) PAM in the genome to rule out additional potential binding sites. If other nonspecific targets were found, the next CCN was selected as the PAM sequence, and the previous step was repeated. Then, the 20 bp after the PAM sequence were regarded as the sgRNA base-pairing region. The secondary structure of the RNA derived from the sgRNA base-pairing region and the dCas9 handle region was predicted using the UNAFold web server. If the RNA was predicted to form the correct hairpin structures, the sgRNA base-pairing region included in the corresponding primer would be synthesized, and the primer was named xxx-F, in which xxx was the name of the matching gene. If the correct hairpin structures were not formed or the strains could not grow, the next CCN was selected as the PAM sequence. The previous steps were repeated until an appropriate sgRNA was found. The sequence of the dCas9 handle region is GTTTTAGAGCTAGAAATAGCAAGTTAAAATAAGGCTAGTCCG. All primers used in this work were synthesized by Genewiz (Suzhou, China) and are listed in Additional file 2: Table S2.

### Plasmid construction

The single gene inhibition plasmids and the multiple gene expression plasmids were constructed from the plasmid pJMP2. For the single gene interference plasmids, the different sgRNA fragments were amplified from pJMP2 by corresponding primers, digested with Acc65I and PmeI, and then ligated into the pJMP2 backbone that was digested with BsrGI and PmeI. For the multiple gene interference plasmids, the different sgRNA fragments were amplified from pJMP2 by the corresponding primers and assembled by the BM Seamless Cloning Kit, which was purchased from Biomed (Beijing, China), as shown in Fig. 5a.

### RT-qPCR assay

The total RNAs of different strains were extracted from the 24 h fermentation broth using the RNAprep Pure Cell/Bacteria Kit. Reverse transcription was performed with the total RNAs as the templates using the FastQuant RT Kit (with gDNase). The transcription levels of genes were detected by quantitative real-time PCR (qRT-PCR) with a Roche LightCycler 480. The relative transcription level of the target gene was quantified by the 2^−ΔΔCT^ [34] method using the *ccpA* gene as the internal control and BS168NU-Sd as the calibrator. Each qPCR run was performed in a 20 μL volume containing 10 μL of 2× SuperReal PreMix Plus, 0.6 μL of each primer, 1 μL of template cDNA and 8.2 μL of RNase-free H_2_O. The amplification conditions were as follows: preheating at 95 °C for 5 min followed by 40 cycles of 95 °C for 5 s, 55 °C for 20 s, and 72 °C for 20 s. Three technical replicates were carried out for each target gene. All reagents for qPCR were from TianGen Biotech (Beijing) Co., Ltd.

### Quantification of surfactin by RP-HPLC

One millilitre of fermentation broth was centrifuged for 10 min at 5000×*g*, and the supernatant was removed to a clean 1.5 mL centrifuge tube. The insoluble substances were removed by filtration through a 0.2 μm filter membrane (Jinlong, Tianjin, China). The concentration of surfactin was analysed by a Waters 2695 HPLC system composed of an autosampler and an UV detector (Waters, USA). A 10 μL aliquot was injected into a Symmetry^®^ C18 column (5 μm, 250 × 4.6 mm) (Waters, USA) to separate the surfactin isoforms. A surfactin standard (purity ≥ 98%, Sigma-Aldrich Trading Co. Ltd., Shanghai, China) was used to confirm the identity of the fractions. The mobile phases were 10% water and 90% methanol, containing 0.1% trifluoroacetic acid (TFA) with a total flow rate of 1.0 mL/min, and the chromatograms were detected at 205 nm with a column temperature of 30 °C. The total concentration of surfactin was calculated from the total peak area of four surfactin isoforms according to the concentration standard curve obtained by using Sigma surfactin.

## Results and discussion

### Establishment of surfactin biosynthesis in *B. subtilis* 168

The wild-type *B. subtilis* 168 strain (BS168NU) is incapable of synthesizing surfactin since there is a termination codon in the middle of the *sfp* gene, which encodes the phosphopantetheinyl transferase (PPTase) that plays an essential role in surfactin synthesis [35]. Thus, we integrated the complete *sfp* gene into the genomic *ydeO* site of the wild-type *B. subtilis* 168 strain, which generated the recombinant BS168NU-S strain. The surfactin production by the BS168NU-S strain was 0.45 g/L, which was determined 24 h after inoculation in 5 mL of semi-defined fermentation medium with sucrose as the carbon source (Additional file 3: Figure S1).

### Construction of the CRISPRi system and the relative expression level analysis

**Fig. 2:**
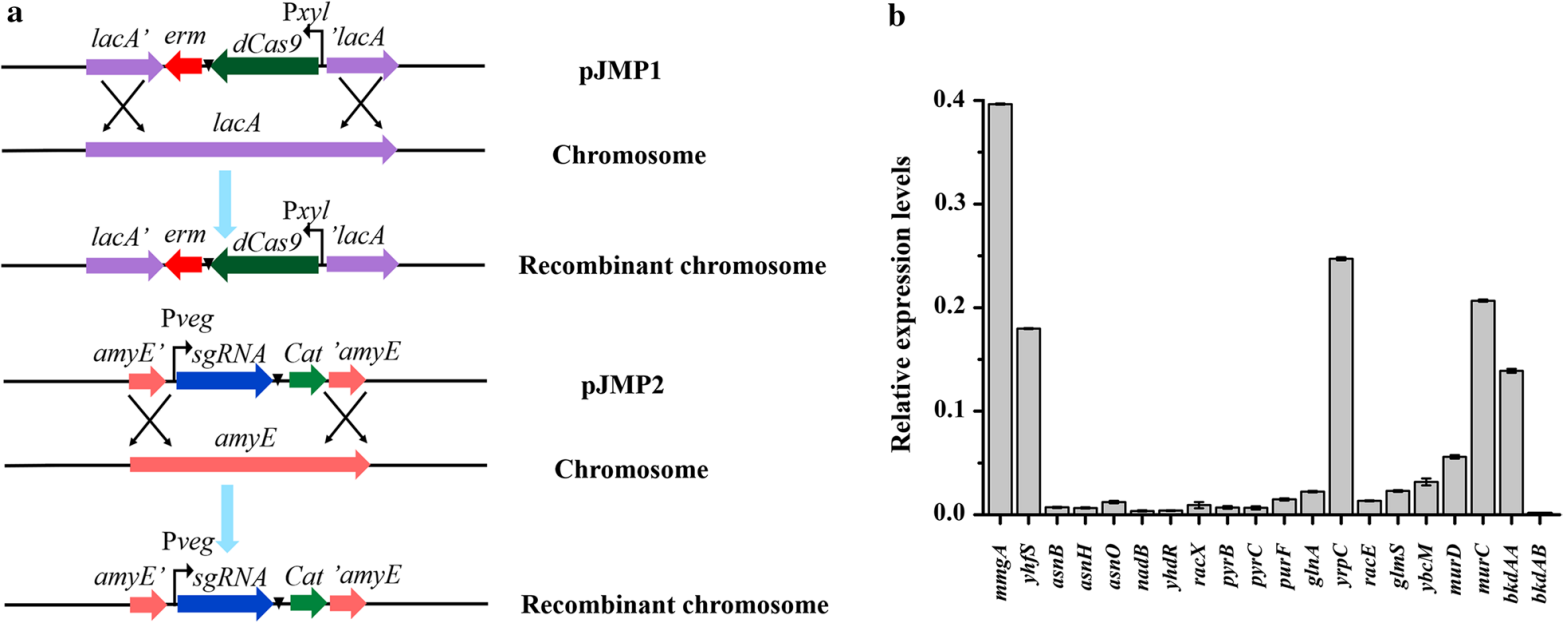
Construction of the CRISPRi system and assay of the relative expression levels. **a** Schematic diagram of the integration of dCas9 and all sgRNA expression cassettes in the *B. subtilis* strain BS168NU-S. The dCaS9 cassette, which was expressed under the control of the inducible promoter of P*xyl*, was integrated into the *lacA* site of the genome. The sgRNA was expressed by the constitutive promoter of P*veg* and integrated into the *amyE* site of the genome. **b** Characterization of the expression levels of the inhibited genes through qRT-PCR at 24 h. The relative transcription level of the target gene was quantified by the 2^−ΔΔCT^ method using the *ccpA* gene as the internal control and BS168NU-Sd as the calibrator

Many efforts have been made to enhance surfactin production, including increasing the of fatty acid supply [17] and overexpressing surfactin synthase [8, 9, 12, 17]. Here, we attempted to improve surfactin biosynthesis by increasing the supply of amino acid precursors, which was achieved by inhibiting the branch pathways of amino acid biosynthesis. Twenty genes on the branch pathways of amino acid biosynthesis were selected, including *mmgA*, *yhfS*, *nadB*, *yhdR*, *asnB*, *asnH*, *asnO*, *racX*, *pyrB* and *pyrC* (on the branch pathways of l-aspartate biosynthesis), *purF*, *glnA*, *yrpC*, *racE*, *glmS*, *ybcM*, *murD* and *murC* (on the branch pathways of l-glutamate biosynthesis), and *bkdAA* and *bkdAB* (on the branch pathways of l-valine and l-leucine biosynthesis) (Fig. 1b). First, these genes interfered individually with the CRISPRi system. Previous studies indicated that in *B. subtilis* 168, the expression of dCas9 by the P*xyl* promoter could achieve higher inhibition efficiency than the expression of dCas9 by other promoters [36, 37]. Thus, in this study, dCas9 was expressed under the control of the inducible P*xyl* promoter and was integrated into the *lacA* site of the *B. subtilis* genome. The sgRNA was expressed by the constitutive P*veg* promoter and integrated into the *amyE* site of the genome (Fig. 2a). To evaluate the silencing efficiency of the CRISPRi system in *B. subtilis*, the transcription levels of the 20 genes were detected by quantitative real-time PCR (qRT-PCR) with a Roche LightCycler 480. The cDNAs were obtained by reverse transcription with the total RNA extracted from 24 h fermentation broth as the template. The relative transcription level of the target gene was quantified by the 2^−ΔΔCT^ [34] method using the *ccpA* gene as the internal control and BS168NU-Sd as the calibrator. The results proved that the CRISPRi system effectively inhibited gene expression in *B. subtilis* 168 (Fig. 2b). However, the repression efficiencies of different genes varied. The transcription repression efficiency of *mmgA*, *yhfS*, *yrpC*, *murC* and *bkdAA* genes ranged from 2.5- to 7.2-fold. The transcription levels of other genes all exhibited over tenfold repression. In particular, significant decreases in the transcriptional levels of *asnH*, *nadB*, *yhdR*, *pyrC* and *bkdAB* genes (over 150-fold) were observed, which was consistent with a previous study [37].

### Effect of single gene inhibition on surfactin production

**Fig. 3:**
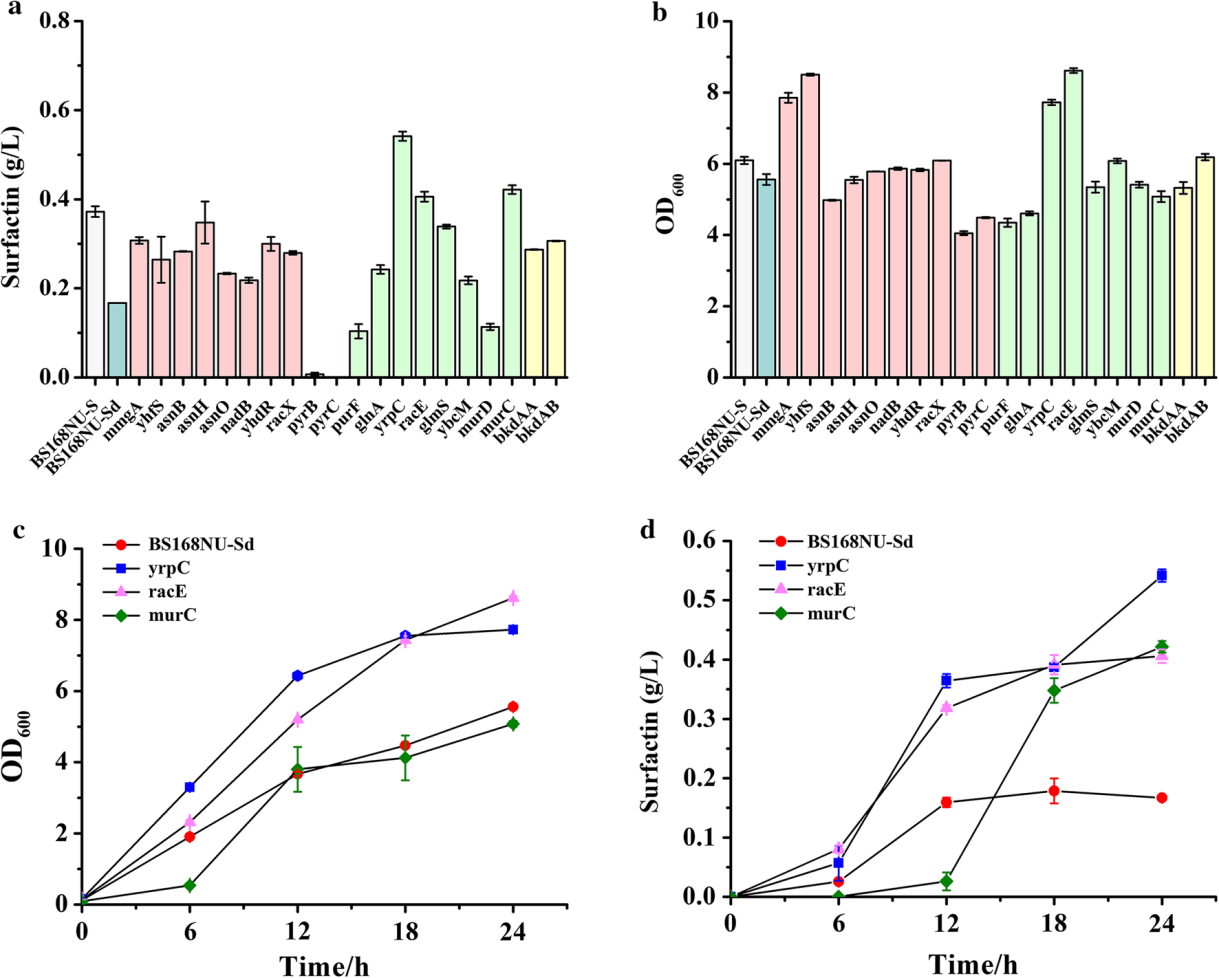
Effect of single gene inhibition on surfactin production in *B. subtilis*. **a** The surfactin production resulting from CRISPRi-based single gene interference in the BS168NU-Sd strain with the addition of 4 g/L xylose as an inducer. **b** The OD_600_ of single gene interference in the BS168NU-Sd strain with the addition of 4 g/L xylose inducer in the medium. **c** Changes in OD_600_ in the BS168NU-Sd, yrpC, racE and murC strains. **d** Changes in surfactin production in the BS168NU-Sd, yrpC, racE and murC strains. The surfactin production and cell growth of the recombinant strains were all determined 24 h after inoculation in 5 mL of semi-defined fermentation medium. The different colours represent the different types of strains: light grey: wild type; green: control; light cameo brown: the strains with l-aspartate metabolic modules; light yellow: the strains with l-valine and l-leucine metabolic modules; light green: the strains with l-glutamate metabolic modules

First, BS168NU-Sd was constructed as the control by integrating the *dCas9* gene into the *lacA* site of the BS168NU-S genome without the sgRNA cassette. Surfactin production and cell growth were determined 24 h after inoculation in 5 mL of semi-defined fermentation medium with the addition of 4 g/L xylose inducer (Fig. 3a, b). The surfactin titre of BS168NU-Sd was only 0.17 g/L, which was lower than that of BS168NU-S (0.37 g/L). This negative impact on surfactin production resulted from the toxicity of the highly expressed dCaS9 protein [38]. Despite this effect, however, the individual inhibition of 16 among the 20 selected genes could increase the production of surfactin. In particular, when the gene *yrpC*, *racE* or *murC* was inhibited, the surfactin production was increased to 0.54, 0.41 or 0.42 g/L, respectively. The *murC*-inhibited strains also had the highest surfactin production per OD_600_ of 0.083 g/L/OD_600_ (Additional file 4: Figure S2). These three genes are related to the consumption of l-glutamate [39] (Fig. 1b), whose acylation is the first step in surfactin assembly.

To evaluate the differences over time, the surfactin production and cell biomass of BS168NU-Sd, yrpC, racE and murC were measured 0 h, 6 h, 12 h, 18 h, and 24 h after inoculation (Fig. 3c, d). The growth of the *yrpC*- and *racE*-inhibited strains was greater than that of the parent BS168NU-Sd strain throughout the fermentation (Fig. 3c). YrpC and RacE are MurI-type glutamate racemase [40], which not only catalyses the conversion of l-glutamate to d-glutamate [39] but also is a DNA gyrase inhibitor [40, 41]. Overexpression of these MurI-type glutamate racemases caused growth retardation [40–42] because of the repression of DNA gyrase activity, which plays an essential role in DNA supercoiling and cell replication [43, 44]. Thus, it was predicted that the inhibition of *yrpC* and *racE* could increase the growth rate, and the results supported this assumption, as shown in Fig. 3c. However, although the growth of the *murC*-inhibited strain had a longer lag period and slightly less final biomass than BS168NU-Sd, both the maximum growth rate and the volumetric productivity were higher than those of BS168NU-Sd (Fig. 3c and Table 3). Usually, high productivity is expected to lower cell growth, since cells might redirect energy from growth to production. However, the phenomena that engineered strains could show both higher biomass and higher production were also observed in previous studies on surfactin biosynthesis [8, 45] and might be caused by a higher central metabolism or higher substrate utilization rate in the engineered strains. The three engineered strains indeed used more sucrose than BS168NU-Sd, as shown in Additional file 5: Figure S3.

**Table 3:** The production, volumetric productivity and yield of the parent strains and the recombinant strains with racE, murC, yrpC, H1, H2, and H3

Strains	Target genes	Titre (mg/L)	Volumetric productivity (mg/L/h)	Yield on carbon (mmol/mol sucrose)
BS168NU-Sd	–	166.88	6.95	0.80
BS168NU-S	–	372.30	15.51	1.75
racE	*racE*	405.83	16.91	1.93
murC	*murC*	421.58	17.57	1.98
H3	*racE*, *murC*	478.69	19.95	2.26
yrpC	*yrpC*	541.57	22.57	2.55
H2	*yrpC*, *murC*	566.41	23.60	2.69
H1	*yrpC*, *racE*	751.90	31.33	3.54

The *mmgA*- and *yhfS*-inhibited strains also showed clear increases in cell biomass. The genes *mmgA* and *yhfS* encode the acetyl-CoA C-acetyltransferase, which catalyses acetoacetyl-CoA biosynthesis from acetyl-CoA. The inhibition of *mmgA* and *yhfS* could reduce the consumption of acetyl-CoA, a key intracellular intermediate metabolite, which is not only used for surfactin biosynthesis but also benefits cell growth and proliferation [46].

In contrast, the strain with inhibition of *pyrB* or *pyrC*, which catalyses the conversion of l-aspartate to uracil, produced negligible amounts of surfactin. These results were consistent with previous research showing that the disruption of *pyrB* and *pyrC* decreased surfactin production [47], which indicated that high inhibition of these genes might be harmful to surfactin biosynthesis. Thus, we decreased the repression intensity by using the leaked expression of the dCas9 protein by the P*xyl* promoter [37]. As expected, the *pyrB*- or *pyrC*-inhibited strains exhibited surfactin production of 0.34 or 0.36 g/L when no xylose was added (Additional file 6: Figure S4), which were much higher values than that obtained when dCas9 was induced by 4 g/L xylose (Fig. 3a). The results indicated that sometimes the gene repression efficiency needs to be delicately regulated to improve the biosynthesis of desired products.

### Inhibition of *bkdAA* and *bkdAB* changed the composition of surfactin

**Fig. 4:**
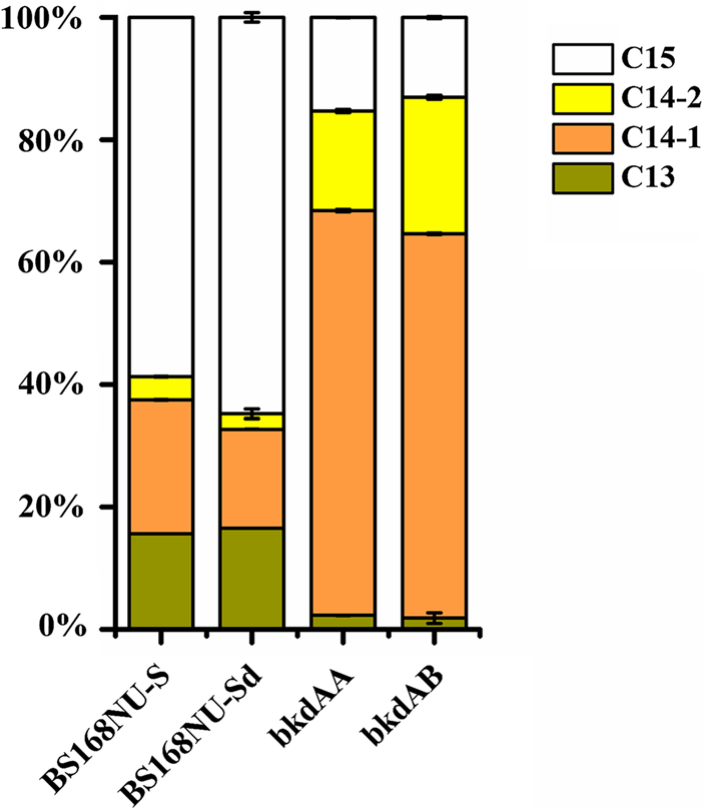
Effect of inhibiting *bkdAA* and *bkdAB* on the composition of surfactin. Cn represents the carbon chain lengths of β-hydroxy fatty acids, where n is the number of carbon atoms. The amino acid sequence of the heptapeptide is Glu-Leu-Leu-Val-Asp-Leu-Leu, except for C_14_-2, which has the sequence Glu-Val-Leu–Leu-Asp-Leu-Val

Surfactin is a mixture in terms of the length of the fatty acid carbon chain and the structural content of the peptide moiety. Razafindralambo et al. [48] found that the foaming capacity and quality of C_14_ surfactin were higher than those of C_13_ and C_15_ surfactin. The inhibition of *bkdAA* or *bkdAB* not only increased surfactin production compared with that of BS168NU-Sd but also significantly changed the proportion of each component of surfactin [20]. The proportion of C_14_ in the *bkdAA*- or *bkdAB*-inhibited strain increased from 25.7% (BS168NU-S) or 18.7% (BS168NU-Sd) to 82.4% or 85.1%, respectively (Fig. 4, and Additional file 7: Figure S5) [49]. These results might occur because the inhibition of *bkdAA* and *bkdAB* not only increased the accumulation of l-leucine and l-valine, but also decreased the synthesis of iso-C_13_ and iso-C_15_ fatty acids by interrupting the *bkd* operon (*lpdV*, *bkdAA*, *bkdAB* and *bkdB* genes). The *bkd* operon catalyses the synthesis of iso-C_13_ and iso-C_15_ fatty acids using l-leucine as the precursor [49] (Additional file 8: Figure S6).

### Multiple gene inhibition further improved surfactin production

**Fig. 5:**
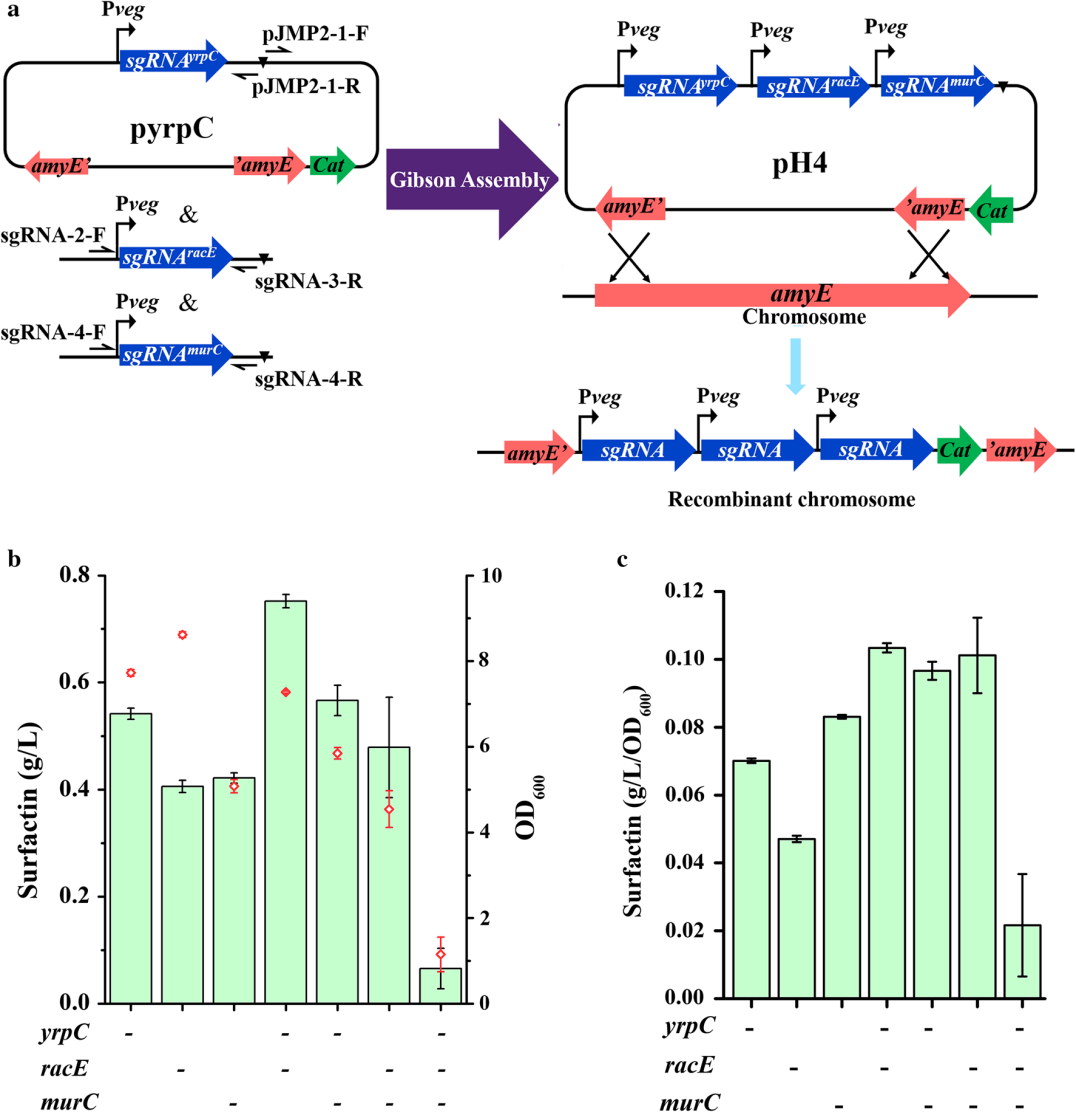
Effect of multiple gene inhibition on surfactin production in *B. subtilis*. **a** Schematic diagram of the construction for multiple gene inhibition by CRISPRi. **b** Surfactin production and OD_600_ of the recombinant strains in which the *yrpC*, *racE* and *murC* genes were inhibited in combination. The histogram represents the production of surfactin, and the scatter represents OD_600_. **c** The surfactin production per OD_600_ of the strains in which *yrpC*, *racE* and *murC* were inhibited in combination. The surfactin production and the cell growth of the recombinant strains were all determined 24 h after inoculation in 5 mL of semi-defined fermentation medium. “-” indicates the inhibition of a specific gene by CRISPRi

According to the results of single gene inhibition, the three genes (*yrpC*, *racE* and *murC*) that most significantly increased surfactin production were selected, and their coherent co-inhibition effects on surfactin production were further studied. The sgRNA plasmids for multiple gene inhibition were constructed as shown in Fig. 5a. The surfactin production and the cell growth of the recombinant strains with multiple gene inhibition were also determined 24 h after inoculation in 5 mL of semi-defined fermentation medium with 4 g/L xylose inducer (Fig. 5b). The results indicated that the simultaneous co-inhibition of two genes, i.e., *yrpC* and *racE*, *yrpC* and *murC*, or *racE* and *murC*, further increased surfactin production to 0.75, 0.57 or 0.48 g/L, respectively (as shown in the histogram of Fig. 5b). The surfactin production per OD_600_ of these double-gene inhibited strains was also increased compared to that of strains with the inhibition of a single gene (Fig. 5c). The co-inhibition of *yrpC* and *racE* produced the highest titre of surfactin (0.75 g/L). Unlike *murC*, the genes *yrpC* and *racE* directly participate in the consumption of l-glutamate, and thus the co-inhibition of *yrpC* and *racE* might cause a higher accumulation of l-glutamate. However, further inhibition of the three genes led to a surfactin titre of only 0.07 g/L, which was lower than that of single gene inhibition. This result may be caused by the significant deterioration in cell growth upon co-inhibition of these three genes (as shown in the scatter of Fig. 5b) [47].

In summary, we improved the capacity of surfactin production in *B. subtilis* by repression of the genes on the branch metabolic pathways of the biosynthesis of 4 amino acid precursors for surfactin synthesis. The production, volumetric productivity and yield of the 6 recombinant strains with the clearest improvement in surfactin synthesis are presented in Table 3. The surfactin production and volumetric productivity of the *yrpC*- and *racE*- inhibited strain were 4.51-fold that of BS168NU-Sd, and the yield on sucrose was 4.43-fold that of BS168NU-Sd. The surfactin production, volumetric productivity and yield of this recombinant strain on sucrose were also more than 2 times higher than those of BS168NU-S.

## Conclusions

To the best of our knowledge, this study is the first to systematically investigate the regulatory effect of increasing the supply of amino acids on surfactin production. First, we constructed an efficient CRISPRi system in *B. subtilis*, which was proven by transcriptional repression as measured by RT-qPCR. Then, 20 core genes on the branch metabolic pathways of four amino acid biosynthesis pathways (i.e., l-glutamate, l-aspartate, l-leucine and l-valine) were individually inhibited by CRISPRi in BS168NU-S. The inhibition of 16 genes increased surfactin production. Among them, the inhibition of *yrpC*, *racE* or *murC,* which are related to l-glutamate metabolism, increased surfactin production most significantly, with titres of 0.54, 0.41 or 0.42 g/L, respectively. Furthermore, the three genes were repressed in combination, and the strain with co-inhibition of *yrpC* and *racE* produced the highest surfactin titre of 0.75 g/L (the specific productivity and yield were 31.33 mg/L/h and 3.54 mmol/mol sucrose, respectively). This work indicated that inhibition of the branch pathways through the CRISPRi system to increase the amino acid precursor supply is an effective strategy for improving surfactin production in *B. subtilis.*

## Additional files

**Additional file 1: Table S1.** The sequences of *sfp* and *dCas9*.

**Additional file 2: Table S2.** Primer sequences used in this study.

**Additional file 3: Figure S1.** Construction of the surfactin production *B. subtilis* strain BS168NU-S. (A) Schematic diagram of the double crossover homologous recombination for construction of the recombinant *B. subtilis* strain BS168NU-S. Phosphopantetheinyl transferase (PPTase), which plays an essential role in surfactin synthesis, is encoded by the *sfp* gene. However, *sfp* in the wild-type *B. subtilis* 168 strain (BS168NU) is inactive due to a termination codon in the middle of the gene sequence. We thus integrated the *sfp* gene under the control of the P43 promoter into the *ydeO* site of the BS168NU genome using double crossover homologous recombination and the Spizizen transformation approach. (B) Surfactin production by the *B. subtilis* strains of BS168NU and BS168NU-S 24 h after inoculation in 5 mL of semi-defined fermentation medium without xylose inducer.

**Additional file 4: Figure S2.** The surfactin production per OD_600_ of parent strains and with CRISPRi-based single gene interference in the BS168NU-Sd strain 24 h after inoculation in 5 mL of the semi-defined fermentation medium.

**Additional file 5: Figure S3.** Residual sucrose concentrations of BS168NU-Sd, yrpC, racE and murC strains during fermentation.

**Additional file 6: Figure S4.** Surfactin production of the BS168NU-S, BS168NU-Sd, pyrB, and pyrC strains in medium without xylose inducer.

**Additional file 7: Figure S5.** HPLC analysis of surfactin. Peak-1 contains a C_13_-β-hydroxy fatty acid chain and a Glu-Leu-Leu-Val-Asp-Leu-Leu peptide. Peak-2 contains a C_14_-β-hydroxy fatty acid chain and a Glu-Leu-Leu-Val-Asp-Leu-Leu peptide. Peak-3 contains a C_14_-β-hydroxy fatty acid chain and a Glu-Val-Leu-Leu-Asp-Leu-Val peptide. Peak-4 contains a C_15_-β-hydroxy fatty acid chain and a Glu-Leu-Leu-Val-Asp-Leu-Leu peptide [50, 51].

**Additional file 8: Figure S6.** Schematic diagram of the biosynthesis of iso-C_13_ and iso-C_15_ fatty acids using l-leucine as a precursor.

## Data Availability

Data will be made available from the corresponding author on reasonable request.
