# Supplementary material for: Enhancing surfactin production by using systematic CRISPRi repression to screen amino acid biosynthesis genes in Bacillus subtilis
Source: Microb Cell Fact. 2019 May 23;18:90. doi: 10.1186/s12934-019-1139-4 (PMC6533722; doi:10.1186/s12934-019-1139-4)
Supplement: Supplementary file 2 — Additional file 2: Table S2. Primer sequences used in this study. [file 12934_2019_1139_MOESM2_ESM.docx]

**Table S2. Primer sequences**

| Name | Sequence |
| --- | --- |
| ydeO-U1 | GTGTGCTGATATAAAGAGAGTG |
| ydeO-U1-2 | CCAGCCAATCCTCAAGTC |
| ydeO-U2 | AAGCGATACGATTCCGATAA |
|  |  |
| SFP-1 | TTATCGGAATCGTATCGCTTATGACAAACATCACCCTCTT |
| SFP-2 | TGGAATTGTGAGCGGATAA |
| ydeO-D1 | TTATCCGCTCACAATTCCACGATGAAGGTCTGTATCACA |
| ydeO-G2 | GCTACATCATACACGGTAAC |
| sgRNA-R | CTTCTCTCATCCGCCAAAAC |
| yhfs-F | CCCGGTACCATAAATGTgccaaatcctccggcaggaaGTTTTAGAGCTAGAAATAGC |
| mmgA-F | CCCGGTACCATAAATGTactccgccgaatttgccaaaGTTTTAGAGCTAGAAATAGC |
| glnA-F | CCCGGTACCATAAATGTgaatgtcagtaaattgaaggGTTTTAGAGCTAGAAATAGC |
| nadB-F | CCCGGTACCATAAATGTgggcgggaaagctgcagctaGTTTTAGAGCTAGAAATAGC |
| asnH-F | CCCGGTACCATAAATGTaaggtacattcctgagaacgGTTTTAGAGCTAGAAATAGC |
| asnO-F | CCCGGTACCATAAATGTtgtccatcgtttgtttttccGTTTTAGAGCTAGAAATAGC |
| asnB-F | CCCGGTACCATAAATGTtgatcagcggtttgagctaaGTTTTAGAGCTAGAAATAGC |
| racX-F | CCCGGTACCATAAATGTggtcgatttcggccccattcGTTTTAGAGCTAGAAATAGC |
| pyrB-F | CCCGGTACCATAAATGTtcgtgctcggttcgaaaaacGTTTTAGAGCTAGAAATAGC |
| pyrC-F | CCCGGTACCATAAATGTtgatggtttctccagtcactGTTTTAGAGCTAGAAATAGC |
| ybcM-F | CCCGGTACCATAAATGTtactgtacttcagctcgccgGTTTTAGAGCTAGAAATAGC |
| purF-F | CCCGGTACCATAAATGTaccgtaatacgtgatttgcgGTTTTAGAGCTAGAAATAGC |
| glmS-F | CCCGGTACCATAAATGTtccggctttcgcttctacatGTTTTAGAGCTAGAAATAGC |
| bkdAB-F | CCCGGTACCATAAATGTttcttcctacatcttccccaGTTTTAGAGCTAGAAATAGC |
| bkdAA-F | CCCGGTACCATAAATGTtcagccctagtgcttgatgtGTTTTAGAGCTAGAAATAGC |
| yrpC-F | CCCGGTACCATAAATGTttcataaggaagaacttttaGTTTTAGAGCTAGAAATAGC |
| racE-F | CCCGGTACCATAAATGTccggaatcaatgactcctatGTTTTAGAGCTAGAAATAGC |
| murD-F | CCCGGTACCATAAATGTatccgcttttcgcaagcccaGTTTTAGAGCTAGAAATAGC |
| murC-F | CCCGGTACCATAAATGTtcatgaagtatttgggcaagGTTTTAGAGCTAGAAATAGC |
| sgRNA-CE-F | GCGATTTCCAATGAGGTTAA |
| sgRNA-CE-R | GGAGACCGTTTAAACTCAATG |
| pJMP2-1-F | CCCGAACAAAAACTCATCTCA |
| pJMP2-1-R | CAAAAAAAGCACCGACTCGG |
| sgRNA-2-F | CCGAGTCGGTGCTTTTTTTGCTTATTAACGTTGATATAAT |
| sgRNA-3-R | TGTACAACTAGTCCGCACCGACTCGGTGCCACTT |
| sgRNA-4-F | GGACTAGTTGTACACTTATTAACGTTGATATAAT |
| sgRNA-4-R | TGAGATGAGTTTTTGTTCGGGGCACCGACTCGGTGCCACTT |
| dCas9-R | GCACATTTCAAACGAATACGG |
| xylR-F | ATCATTGTCCTGATCCTGC |
| RT-ccpA1 | ACGAGCATGTGGCGGAAT |
| RT-ccpA2 | GATAGCGACTGACGGTGT |
| mmgA-RT-F | GCCGCCGATGAAGGGAAGTTT |
| mmgA-RT-R | TCCTCAGACATCAGCACGAACG |
| yhdR-RT-F | CTCAGCCATCAACGCAGCAGGA |
| yhdR-RT-R | ACTGCTGTTGGCTGCTGTGACT |
| asnB-RT-F | CGAAGAACGGCACGACGAAGT |
| asnB-RT-R | CAGCCTTGTCCGCACAATGGT |
| asnO-RT-F | GCGAGTGGACAGAACGGGACTT |
| asnO-RT-R | AACAGGCACATCCGACACAAGC |
| asnH-RT-F | CGTTCTTGGACCATCGGCTTGT |
| asnH-RT-R | CTGGAGGCATCGCTGTGAGAGA |
| nadB-RT-F | ACGAGGCGGTGTTTGTCTTGGA |
| nadB-RT-R | TCGTCTGCGTGCCTTACCTTGA |
| yhdR-RT-F | ACCCGATTGTTGAACCGCCTGA |
| yhdR-RT-R | ATGACGATGCGCTCCGCTGAGA |
| racX-RT-F | ATTCATGCCCGACGCCCTTCT |
| racX-RT-R | GCGGATTGGATGGTTGGCTCTG |
| pyrB-RT-F | CACGGCGACATCAAGCATAGCA |
| pyrB-RT-R | CCTTCCTGACTGACAGCGGACT |
| pyrC-RT-F | ACGGGCTAAACGGCATTCCTTC |
| pyrC-RT-R | TGCGGCGATACTTCTGCTGTCA |
| purF- RT-F | GCAGAGGCAACAGGCATTCCGT |
| purF-RT-R | CGCACCGCAGACAGCTTCATTC |
| glnA-RT-F | AAGGCGAGCCGACGCTTGAA |
| glnA-RT-R | TCGTGCTGACCAGGTGCTACTT |
| yrpC-RT-F | TCAGGAATTGGTGGCATGACTG |
| yrpC-RT-R | TAGCAGGCTTTACAGCGGGTTC |
| racE-RT-F | ACATCCAGCGCAGCGTCGGTAT |
| racE-RT-R | AAGCAGCGGGCAGGCAAGGTTT |
| glmS-RT-F | ATGCGATGGCGATGCTTCAAGT |
| glmS-RT-R | AACAACAGGCTGCTCATCCGTT |
| ybcM-RT-F | GCGGCGAGCTGAAGTACAGTAC |
| ybcM-RT-R | CGACAACGGAAACAGAGCAAGC |
| murD-RT-F | ACATTCTGGCGACAAGCAAGGC |
| murD-RT-R | GCGGGTGACAGCAGGATGACAT |
| murC-RT-F | CGCATTCCCAGACACGCATCCT |
| murC-RT-R | CGGTATTCGCACGCTTCAAACAC |
| bkdAA-RT-F | CGAAAGGGCACGCAGAGGAGAA |
| bkdAA-RT-R | TCCGCTTCATCCGTCGCTTCAT |
| bkdAB-RT-F | TGGATACGCCGCTTGCTGAATC |
| bkdAB-RT-R | CGCTCTGACGACAATCGGACAG |
