# Supplementary figures and images for: Enhancing surfactin production by using systematic CRISPRi repression to screen amino acid biosynthesis genes in Bacillus subtilis
Source: Microb Cell Fact. 2019 May 23;18:90. doi: 10.1186/s12934-019-1139-4 (PMC6533722; doi:10.1186/s12934-019-1139-4)

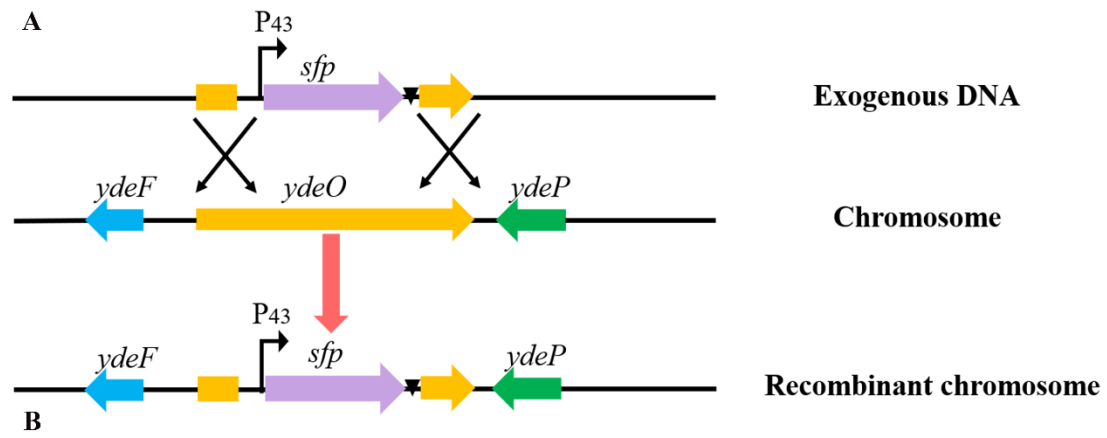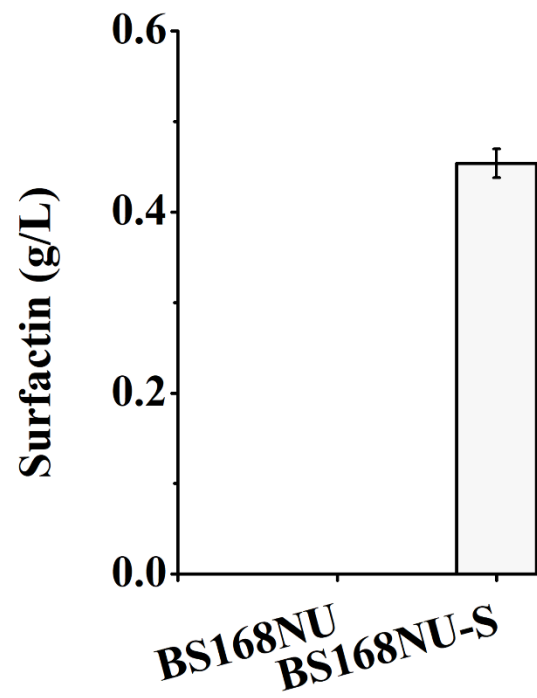

Supplement: Supplementary file 3 — Additional file 3: Figure S1. Construction of the surfactin production B. subtilis strain BS168NU-S. (A) Schematic diagram of the double crossover homologous recombination for construction of the recombinant B. subtilis strain BS168NU-S. Phosphopantetheinyl transferase (PPTase), which plays an essential role in surfactin synthesis, is encoded by the sfp gene. However, sfp in the wild-type B. subtilis 168 strain (BS168NU) is inactive due to a termination codon in the middle of the gene sequence. We thus integrated the sfp gene under the control of the P43 promoter into the ydeO site of the BS168NU genome using double crossover homologous recombination and the Spizizen transformation approach. (B) Surfactin production by the B. subtilis strains of BS168NU and BS168NU-S 24 h after inoculation in 5 mL of semi-defined fermentation medium without xylose inducer. [file 12934_2019_1139_MOESM3_ESM.pdf]

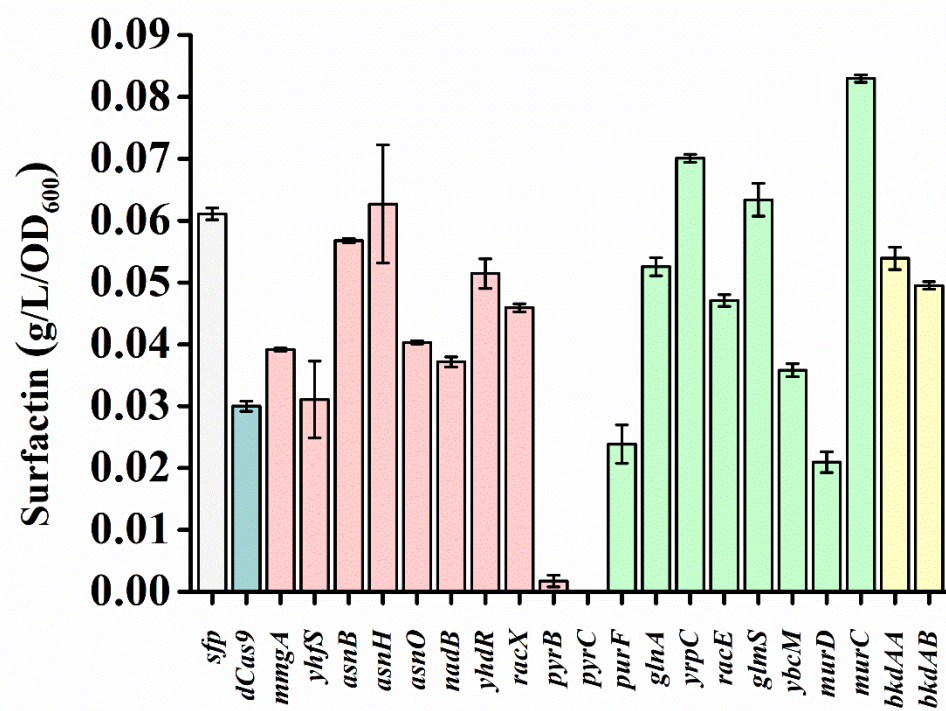

Supplement: Supplementary file 4 — Additional file 4: Figure S2. The surfactin production per OD600 of parent strains and with CRISPRi-based single gene interference in the BS168NU-Sd strain 24 h after inoculation in 5 mL of the semi-defined fermentation medium. [file 12934_2019_1139_MOESM4_ESM.pdf]

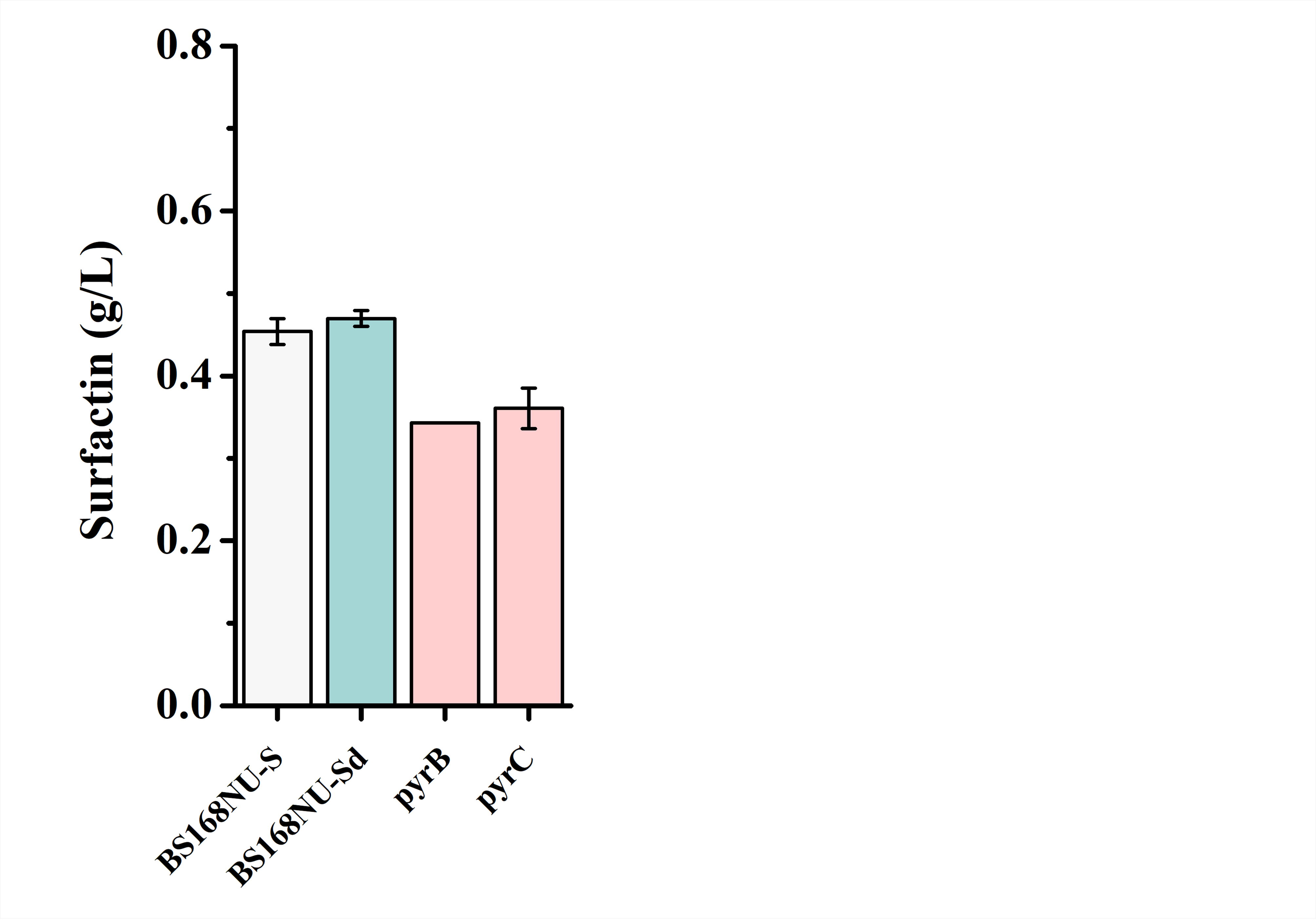

Supplement: Supplementary file 6 — Additional file 6: Figure S4. Surfactin production of the BS168NU-S, BS168NU-Sd, pyrB, and pyrC strains in medium without xylose inducer. [file 12934_2019_1139_MOESM6_ESM.tif]

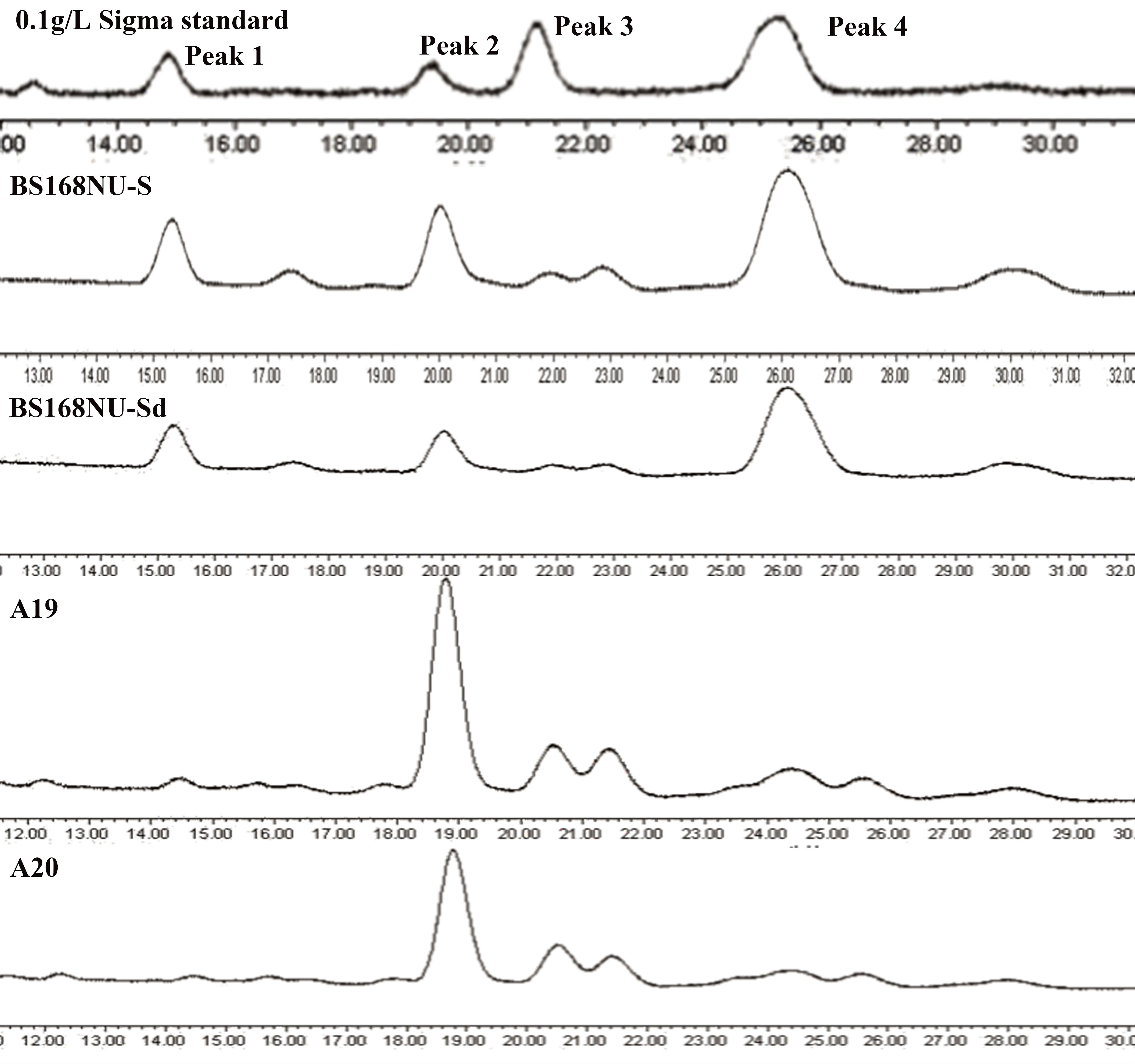

Supplement: Supplementary file 7 — Additional file 7: Figure S5. HPLC analysis of surfactin. Peak-1 contains a C13-β-hydroxy fatty acid chain and a Glu-Leu-Leu-Val-Asp-Leu-Leu peptide. Peak-2 contains a C14-β-hydroxy fatty acid chain and a Glu-Leu-Leu-Val-Asp-Leu-Leu peptide. Peak-3 contains a C14-β-hydroxy fatty acid chain and a Glu-Val-Leu-Leu-Asp-Leu-Val peptide. Peak-4 contains a C15-β-hydroxy fatty acid chain and a Glu-Leu-Leu-Val-Asp-Leu-Leu peptide [50, 51]. [file 12934_2019_1139_MOESM7_ESM.tif]

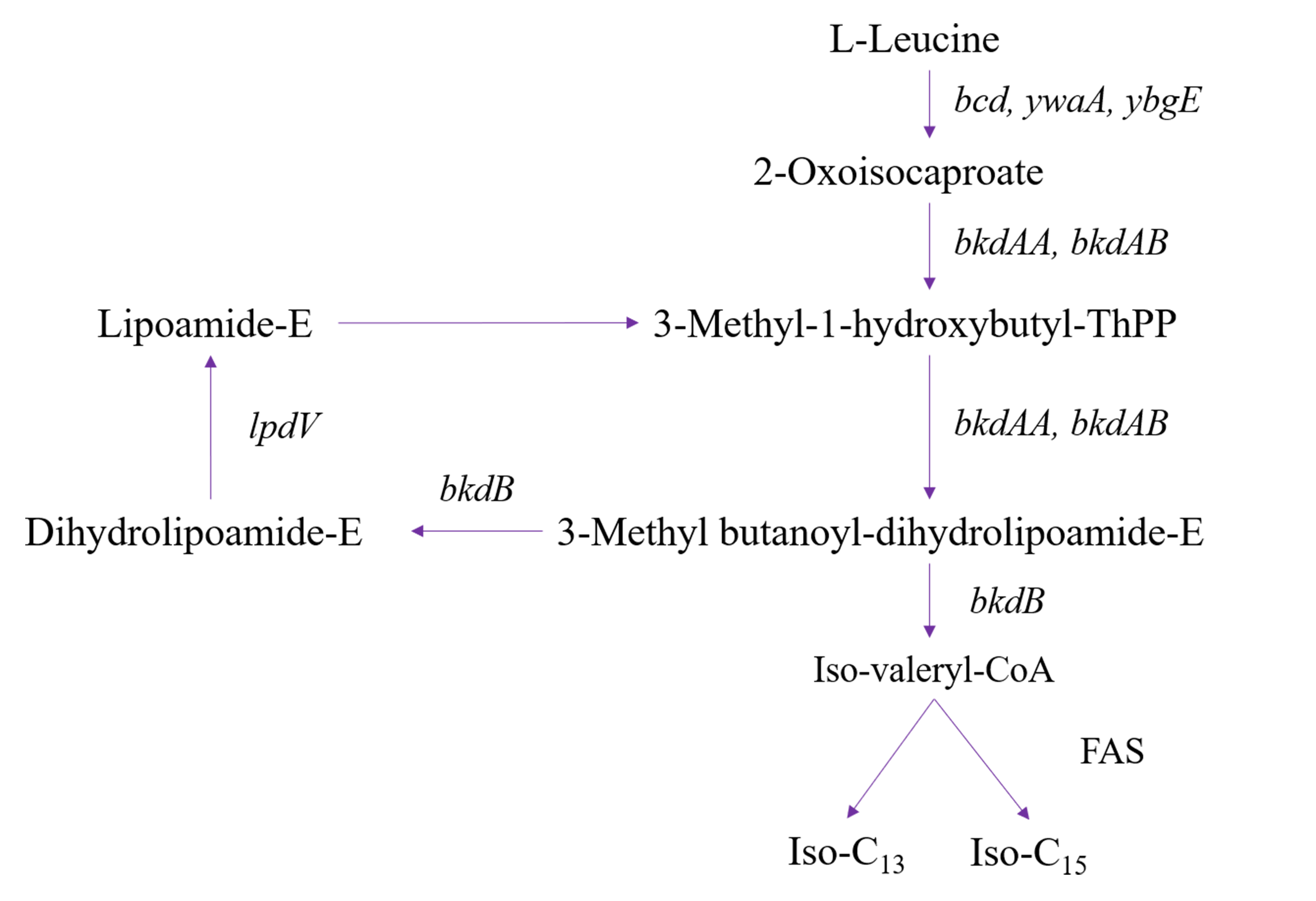

Supplement: Supplementary file 8 — Additional file 8: Figure S6. Schematic diagram of the biosynthesis of iso-C13 and iso-C15 fatty acids using l-leucine as a precursor. [file 12934_2019_1139_MOESM8_ESM.tif]
